# Oxalic Acid Preharvest Treatment Improves Colour and Quality of Seedless Table Grape ‘Magenta’ Upregulating on-Vine Abscisic Acid Metabolism, Relative *Vv*NCED1 Gene Expression, and the Antioxidant System in Berries

**DOI:** 10.3389/fpls.2021.740240

**Published:** 2021-11-01

**Authors:** María E. García-Pastor, María J. Giménez, Vicente Serna-Escolano, Fabián Guillén, Daniel Valero, María Serrano, Santiago García-Martínez, Leon A. Terry, M. Carmen Alamar, Pedro J. Zapata

**Affiliations:** ^1^Department of Food Technology, EPSO, University Miguel Hernández, Alicante, Spain; ^2^Department of Applied Biology, EPSO, University Miguel Hernández, Alicante, Spain; ^3^Plant Science Laboratory, Cranfield University, Bedfordshire, United Kingdom

**Keywords:** *Vitis vinifera* L., anthocyanins, plant hormones, ripening, senescence

## Abstract

The effect of oxalic acid (OA) in determining poorly coloured table grape quality remains relatively unknown. Some red cultivars, such as seedless table grape ‘Magenta’ are characterised by a poor berry colour, an attribute highly demanded by the consumer. The aim of this research was to elucidate the effect of a preharvest OA treatment (5 mM) on berry colour and quality of table grape by investigating its role in berry development, on-vine ripening, and postharvest senescence. We found that OA significantly increased abscisic acid (ABA) and ABA glucose ester (ABA–GE) content in treated berries. This increase was mediated by changes in the ABA biosynthetic pathway, specifically by the upregulation of the 9-*cis*-epoxycarotenoid dioxygenase (*Vv*NCED1) gene. The accumulation of ABA in treated berries resulted in colour improvement and a higher individual and total anthocyanins content at harvest compared with control; whereas at harvest, OA-treated table grapes showed a significantly lower glucose and fructose content and a higher content of tartaric, ascorbic, and succinic acids. Furthermore, antioxidant enzyme activity was increased during berry development in OA-treated berries. On the other hand, those berries treated with OA showed a delay in loss of firmness and colour during cold storage, as well as less susceptibility to postharvest decay incidence. This effect of OA delaying the senescence process was also related to enzymatic antioxidant system stimulation. For the first time, the role of OA on increasing quality, mainly colour, in table grapes was elucidated, highlighting that this treatment upregulated ABA metabolism, relative *Vv*NCED1 gene expression and antioxidant system, delaying postharvest berry senescence.

## Graphical Abstract 1

Experimental design for oxalic acid preharvest treatment and main results from a metabolomic approach on increasing colour and quality of table grape.

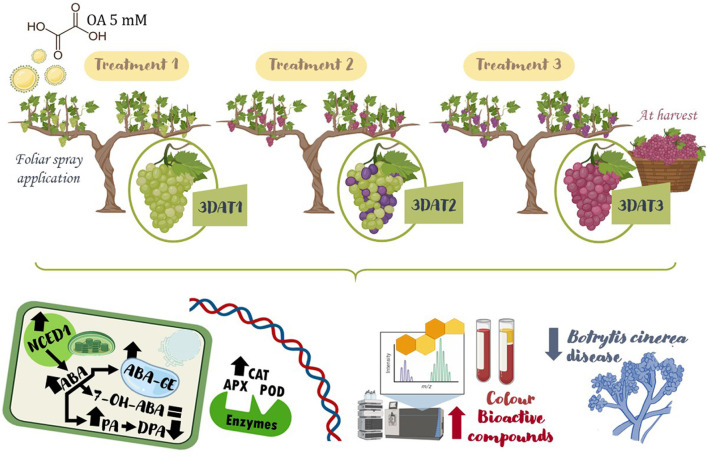

## Introduction

Oxalic acid (OA) is a natural compound that can induce systemic resistance against fungal, bacterial, and viral diseases, through an increase in defence-related enzyme activities and secondary metabolites (Tian et al., 2006; Zheng et al., 2012). Both pre and postharvest OA treatments for extending shelf-life and maintaining the quality of postharvest fruits and vegetables have been investigated and developed for commercial use (Razavi and Hajilou, 2016). Recently, the application of natural and ecofriendly compounds as preharvest treatments has received considerable attention (Martínez-Esplá et al., 2017). In these studies, a delay in ripening and senescence and also the preservation of fruit and vegetable quality were observed. OA has demonstrated an important role in delaying senescence in harvested fruits (Wu et al., 2011), and its effect has been recently reported for sweet cherry (Martínez-Esplá et al., 2014), peach (Razavi and Hajilou, 2016), kiwifruit (Zhu et al., 2016), plum (Martínez-Esplá et al., 2019), and pomegranate fruit (García-Pastor et al., 2020a). However, there is a paucity of research about the effect of OA pre- (Kok and Bal, 2019) and postharvest (Sabir and Sabir, 2017; Hazarika and Marak, 2021) applications on table grape quality.

Grape berries are non-climacteric fruits that display a double sigmoidal growth curve in which the ripening onset is considered as veraison time (Castellarin et al., 2007). Veraison can be identified as the beginning of grape ripening, which is followed by berry softening and increases in skin colour and sugar content. Both, ethylene and abscisic acid (ABA), as well as tehir apparent crosstalk, are likely to be required to initiate berry ripening (Sun et al., 2010). Thus, many studies have shown that ABA concentration increased at the onset of ripening (Wheeler et al., 2009; Sun et al., 2010; Pilati et al., 2017), and trace endogenous ethylene-induced the expression of the *Vv*NCED1 gene, which is involved in ABA biosynthesis (Sun et al., 2010). 9-*cis*-Epoxycarotenoid dioxygenase (NCED) is the enzyme catalysing the first step of this reaction, which produces xanthoxin, the direct C15 precursor of ABA, from the cleavage of 9-*cis*-violaxanthin or 9-*cis*-neoxanthin. Grapevine NCED genes (*Vv*NCED1, *Vv*NCED2, and *Vv*NCED3) encode enzymes that cleave carotenoids to form the phytohormone ABA. NCED genes have been proposed to be implicated in ABA signalling during the ripening initiation in grape berries (Young et al., 2012). Nevertheless, a lack of correlation between free-ABA and NCED codifying genes in table grape berries suggests that compounds derived from the ABA catabolism/conjugation could also be involved in berry ripening (Wheeler et al., 2009; Castellarin et al., 2016).

Skin colour is a key quality attribute for table grapes. Consumers demand high and homogeneously coloured grapes, which in turn reach premium prices at the market and result in higher returns for growers (Peppi et al., 2006). Nevertheless, some red cultivars, such as the seedless table grapes ‘Magenta’, produced through stenospermocarpy mechanism which leads parthenocarpy in the grapes, are characterised by a poor berry colour and a non-uniform colour along with clusters, which depreciates their commercial value (Peppi et al., 2006; García-Pastor et al., 2019, 2020c). ABA and ethephon (2-chloroethylphosphonic acid, which is an ethylene-releasing compound) have been commercially used in viticulture to improve colour homogeneity (Peppi et al., 2006; Alenazi et al., 2019; Koyama et al., 2019). However, the effects of ethephon on colour are inconsistent and can lead to berry softening (Peppi et al., 2006) and reduced shelf-life (Szyjewicz et al., 1984). In addition, the application time and concentration of ABA is a critical factor for the effective improvement of grape skin colour, which may vary depending on the cultivar and area of application. On the other hand, the high cost of ABA has precluded the development of practical applications (Peppi et al., 2006). Pigment accumulation can be influenced by sugar and hormonal crosstalk (Li et al., 2016). A combined ABA (400 μl L^–1^) and sucrose (90 μl L^–1^) treatment applied close to veraison in ‘Crimson Seedless’ grapes was more effective in accelerating the pigmentation process by significantly increasing anthocyanin levels than when ABA or sucrose treatments were applied independently (Olivares et al., 2017). Unfortunately, these treatments also induce berry softening, which is an undesirable attribute for fresh grapes because softer berries are more susceptible to grey mould caused by *Botrytis cinerea*. Recently, our previous works with some natural and eco-friendly compounds, such as salicylic acid (SA), acetyl salicylic acid (ASA), methyl salicylate (MeSa), and methyl jasmonate (MeJa), applied at preharvest on ‘Magenta’ and ‘Crimson’ table grapes improved berry colour and induced fruit resistance against grey mould (García-Pastor et al., 2019; García-Pastor et al., 2020b, c). Thus, the aim of the present study was to assess the potential effect of OA preharvest treatment, applied at three key points of table grapes development, on quality parameters, including berry colour of ‘Magenta’ poorly coloured table grape at harvest and during postharvest cold storage. This effect will be elucidated based on a metabolomic (ABA, ABA catabolites, and antioxidant enzymes) approach and throughout the study of the relative *Vv*NCED1 gene expression about the action or role of OA during berry development, on-vine ripening, and postharvest senescence.

## Materials and Methods

### Preharvest and Postharvest Experimental Design

This study was performed in the 2018-growing season with the ‘Magenta’ seedless table grape (*Vitis vinifera* L.) cultivar in a commercial vineyard in Calasparra (Murcia, Spain). Vines were 8-year old, planted in sandy soil, at 2.5 m × 3 m spacing, and grafted onto Paulsen 1103 rootstocks. A programmed irrigation system consisting of a drip irrigation line per row with three emitters per plant was used; vine water requirements and fertilisers along the growth cycle were supplied through this system. Pruning and thinning were carried out according to standard procedures for table grape crops, and the vines received no fungicide treatment. OA (Sigma-Aldrich, Madrid, Spain, CAS No. 144-62-7) treatments were performed by foliar spray application (1-L per vine at 5 mM, containing 0.5% Tween 20 as surfactant) onto the whole vine canopy, including leaves and clusters. The sprays were carried out with a 15-L backpack sprayer until runoff. This concentration was chosen as the optimum among three concentrations tested (1, 5, and 10 mM) in previous experiments (growing seasons 2016 and 2017). A 5-mM OA treatment was the best in terms of yield, berry maturity quality, and bioactive compounds (data not shown). Control vines were treated with an aqueous solution of 0.5% Tween 20. A completely randomised block design with five replicates of three vines (15 vines) for each treatment was followed. Treatments were applied three times on the same vines, previously labelled with the type of treatment, during the fruit growth and ripening cycle (viz. T1 on June 22: before the onset of veraison, when berry volume was ∼40% of its final one (≅ 2,100 mm^3^, T2 on July 10: at veraison stage, and T3 on July 26: 3 days before the first harvest date). Treatments were performed during favourable weather conditions where rainfall or winds were not forecasted for the following 24 h. From those five replicates, fifteen berries (five berries per vine) were taken from each replicate (75 berries) per treatment at 3DAT1, 3DAT2 and 3DAT3 stages (3 days after the first, second and third treatments). Relative NCED (*Vv*NCED1) gene expression, ABA metabolic profile and content, and antioxidant enzyme activity in the three different key stages of the berry growth and ripening cycle were analysed.

Fifteen homogeneous clusters (five clusters per vine) were harvested from each replicate (five replicates; 75 clusters) and treated at commercial ripening stage according to the characteristic size, colour, and total soluble solid content of this cultivar (160–180 g kg^–1^) at harvest or 3DAT3 stage. The harvest date was the same for the different treatments (3DAT3; i.e., on July 29). These harvested clusters were immediately transported to the laboratory where the first set of 25 clusters per treatment (five clusters per replicate) were used to determine the bioactive compound content, total antioxidant activity (TAA), individual sugars, and organic acids concentration. Another set of clusters were stored at 2°C and 90 of relative humidity (RH) for 0, 15, 30, and 45 days (10 clusters per sampling date; 40 clusters per treatment). Firmness, colour, ABA, catabolite content, and antioxidant enzyme activity evolution during postharvest storage were determined. In order to test the effect of OA preharvest application on the postharvest decay caused by *B. cinerea*, berries were wounded at harvest with a sterile lancet (6 mm in depth) and inoculated according to García-Pastor et al. (2020b), using one set of 120 berries from two clusters of each of the five replicates (10 clusters per treatment). Disease incidence or severity was assessed after 5 days at (25 ± 1)°C and 80–85% of RH.

### Relative *Vv*NCED1 Gene Expression

The RNA was extracted from 0.1 g of freeze-dried table grapes, using the whole fruit (flesh + skin tissues), according to the protocol described by Le Provost et al. (2007) with slight modifications. Briefly, freeze-dried samples were manually grounded with liquid nitrogen and mixed with 1 ml of extraction buffer (2.5% (w/v) CTAB, 2% (w/v) polyvinylpyrrolidone or PVP K-40, 1.0 M Tris–HCl at pH = 8.0, 0.5 M EDTA, and 5.0 M NaCl) containing β-mercaptoethanol at 2%, previously heated to 65°C, and vortexed vigorously for 15 s. The mixture was incubated for 10 min in a water bath at 65°C and mixed by inverting the tubes every 3 min. For purification, chloroform–isoamyl–alcohol (24:1) was added and mixed the same way. Then, the mixture was centrifugated at 10,000 × *g* for 10 min at room temperature. Finally, the supernatant was transferred to a clean Eppendorf tube and mixed, inverted, and incubated overnight at −20°C with 1/3 volume of LiCl 10 M. Next day, the solution was centrifuged at 10,000 × *g* for 10 min at 4°C and the supernatant was discarded. The pellet was washed with 500 μl of cold (−20°C) 80% ethanol and centrifuged at 10,000 × *g* for 5 min at 4°C. The supernatant was removed, and when the pellet was dried out, it was resuspended in 100 μl of RNAse free-water and stored at −80°C.

According to the recommendations of the manufacturer, A DNase treatment was done by using Baseline-ZERO DNase (Epicentre/Lucigen, United States) on the eluted RNA. RNA quantification was carried out by spectrophotometric absorbance using a NanoDrop 2000 and a Qubit 2.0 Fluorometer (Thermo Fisher Scientific, United States). The expression analysis of the *Vv*NCED1 gene was carried out by GenXPro GmbH (Germany). Total RNA (15–40 ng per reaction) was used as the template for the OneStep qPCR reactions. Reverse transcription and qRT-PCR were performed using the MDX025 Low LOD 1-Step qPCR Mix (Meridian/Bioline, United States/Germany), according to the recommendations of the manufacturer. All reactions were carried out in a volume of 12 μl including 6.0 μl low LOD 1-step RT-qPCR reaction mix (2×), 0.15 μl EvaGreen from Jena Bioscience (100 μM), 0.20 μl Primer (fwd and rev dilution of 10 μM each in 0.5× TE buffer), and 0.12 μl MMLV-RT (100× – from MDX025 Low LOD 1-Step qPCR Mix). RNA from five biological replicates and treatments was used as the template for the qPCR reactions.

*Vitis vinifera* NCED 1 gene (*Vv*NCED1; LOC100232942) was amplified using the described primers in Table 1 (Rattanakon et al., 2016). qRT-PCR was performed on a StepOne Thermocycler system (Applied biosystems, Thermo-Fisher). Thermocycler parameters were 10 min at 50°C as RT-Step, followed by 2 min at 95°C for Taq polymerase activation, then 36 cycles of 5 s at 95°C were programmed for denaturation and 30 s at 62°C for annealing and extension according to Serna-Escolano et al. (2021). Additionally, the quality of amplicons was controlled by a melt curve analysis step showing no side products, as can be seen in Table 1. Expression of the *Vv*NCED1 gene was normalised with three endogenous control genes, namely *V. vinifera* actin-7 (ACT; LOC100232866), *V. vinifera* ubiquitin-60S ribosomal protein L40-2 (UBI; LOC100253716), and *Vitis vinifera* glyceraldehyde-3-phosphate dehydrogenase cytosolic (GAPDH; LOC100233024) on the normalization of genes in table grapes (Reid et al., 2006; Pagliarani et al., 2017), as can also be seen in Table 1. Relative *Vv*NCED1 gene expression in the treated fruit was calculated with respect to control fruit using five biological replicates.

**TABLE 1:** Transcriptomic details of primers for the targeted and endogenous control genes.

Gene		Forward and reverse primers	Amplicon length	NCBI reference sequence
NCED1	F	5′-GCAGAGGACGAGAGTGTAAAGGA-3′	130 pb	XM_019216859.1
	R	5′-GCAGAGTAAAAACACATGAAGCTAGTG-3′		
ACT	F	5′-GCCCCTCGTCTGTGACAATG-3′	100 pb	XM_002282480.4
	R	5′-CCTTGGCCGACCCACAATA-3′		
UBI	F	5′-TCTGAGGCTTCGTGGTGGTA-3′	99 pb	XM_002273532.2
	R	5′-AGGCGTGCATAACATTTGCG-3′		
GAPDH	F	5′-CCACAGACTTCATCGGTGACA-3′	70 pb	XM_002263109.3
	R	5′-TTCTCGTTGAGGGCTATTCCA-3′		

### ABA and Catabolite Analyses

Freeze-dried powdered table grape material of whole berry (flesh + skin tissues) was weighed (5.0 ± 0.1 mg) and extracted with 500 μl of precooled (−20°C) methanol:water:formic acid (60:35:5 v/v/v), as described by Müller and Munné-Bosch (2011) with some modifications. The labeled forms of the compounds, namely (−)-5,8′8′8′-d4-abscisic acid (d4-ABA), (+)-4,5,8′,8′,8′-d5-abscisic acid glucose ester (d5-ABA-GE), (±)-5,8′,8′,8′-d4-7′-hydroxy-ABA (d4-OH-ABA), (−)-7′,7′,7′-d3-phaseic acid (d3-PA), and (−)-7′,7′,7′-d3-dihydrophaseic acid (d3-DPA) were added to the mixture as internal standards. ABA and catabolite content were quantified according to Morris et al. (2018) with slight modifications using an LC/MS-MS instrument with an Agilent 1200 series HPLC system (Agilent, Berkshire, United Kingdom) coupled to a Q-Trap 6500 mass spectrometer (AB Sciex, Framingham, MA, United States). The extracts were analysed by injecting 20 μl onto a Phenomenex 3 μm C18 Luna 100 × 2 mm with a guard column at 40°C. The mobile phases were as follows: (A) 2% acetonitrile in 2 mM ammonium formate and (B) 95% acetonitrile in water with 0.1% formic acid, using an increasing gradient of B (2% for 4 min, 16% at 20 min, and 34.5% at 25 min) at a flow rate of 200 μl min^–1^. Deuterated and non-deuterated ABA metabolites: (−)-DPA, (+)-ABA-GE, (−)-PA, and (±)-7′-hydroxy-ABA were obtained from the National Research Council of Canada-Plant Biotechnology Institute (Saskatoon, SK, Canada), and (±)-ABA was purchased from Sigma-Aldrich (Darmstadt, Germany). A 10-point calibration curve ranging from 0.5 to 3,000 μg L^–1^ was used for quantification. Phytohormone concentration was expressed in nmol or pmol g^–1^ dry weight (DW) and was the mean ± SE of five replicates.

### Antioxidant Enzymes Activity

Ascorbate peroxidase (APX), catalase (CAT), and peroxidase (POD) enzyme activities were measured in the whole berry extracts (flesh + skin tissues) obtained by homogenising 1 g of frozen tissue with 5 ml of phosphate buffer 50 mM, pH 6.8, containing 1% (w/v) of polyvinylpyrrolidone (PVP) and ethylenediamine–tetraacetic acid 1 mM. After centrifugation at 10,000 × *g* for 30 min at 4°C, the supernatant was used for the quantification of each replicate in duplicate, as reported elsewhere (García-Pastor et al., 2020b). Antioxidant enzyme activities were expressed as units of enzymatic activity (U min^–1^ g^–1^) of fresh weight (FW) with one enzymatic unit (U) being defined as a 0.01 decrease of ascorbate at 290 and 240 nm min^–1^ for APX and CAT, respectively, and a 0.01 increase of absorbance at 470 nm min^–1^ for POD. Results were the mean ± SE of five replicates.

### Bioactive Compound Content, Total Antioxidant Activity, and Berry Quality Parameters at Harvest

From the 25 clusters (five clusters per replicate) per treatment, berries from 10 clusters (two clusters per replicate) were peeled to separate the skin from the flesh and those from the other 10 replicates were not peeled. Both sample types (whole berry and skin tissue) were frozen in liquid nitrogen, ground, and kept at −80°C until bioactive compound evaluation and TAA analysis were carried out manually and the ground in a mortar and pestle. Ten berries were ground from each replicate (five bunches; 50 berries) and treatment to obtain a homogeneous juice sample for the quantification of total soluble solids (TSS), total acidity (TA), individual sugars, and organic acids. All results were the mean ± SE of five replicates at harvest.

Anthocyanins were extracted from 10 and 1 g of frozen berry and skin tissue with 15 and 5 ml of methanol: formic acid: water (25:1:24, v/v/v), respectively. Then, the samples were sonicated in an ultrasonic bath for 60 min and centrifuged at 10,000 × *g* for 15 min. Total anthocyanin concentration was measured and expressed as García-Pastor et al. (2020c). The supernatant was filtered through a 0.45 μm PVDF filter (Millex HV13, Millipore, Bedford, MA, United States) and used for individual anthocyanin quantification by injecting 20 μl of extract into a high-performance liquid chromatography (HPLC) system (Agilent HPLC1200 Infinity series, Agilent Technologies Inc., Waldbronn, Germany), as previously reported (García-Pastor et al., 2020c). Total and individual anthocyanins results were expressed in mg 100 g^–1^ FW.

Total phenolics were extracted by using 5 and 1 g of frozen berry and skin tissue with 10 and 5 ml of water:methanol (2:8) containing 2 mM NaF (to inactivate polyphenol oxidase activity and prevent phenolic degradation), respectively, and then, phenolics were quantified in the supernatant using the Folin–Ciocalteu reagent, as previously reported (García-Pastor et al., 2020c). Results were expressed as mg gallic acid equivalent 100 g^–1^ FW.

To measure TAA, 5 and 1 g of frozen berry and skin tissue, respectively, were homogenised with 5 ml of 50 mM phosphate buffer pH = 7.8 and 5 ml of ethyl acetate. As previously described by García-Pastor et al. (2020a), hydrophilic (H-TAA) and lipophilic (L-TAA) TAA were determined and measured in duplicate in each extract using a reaction mixture in which ABTS^+^ radicals are generated and monitored at 730 nm. Results were expressed as milligrams of Trolox equivalent (TE) 100 g^–1^ FW.

The TSS content was determined in duplicates with a digital refractometer at Atago PR-101 (Atago Co., Ltd., Tokyo, Japan) at 20°C, and expressed as g 100 g^–1^. TA was determined also in duplicates in the same juice by automatic titration (785 DMP Titrino, Metrohm) with 0.1 N NaOH up to pH 8.1, and the results were expressed as grams of tartaric acid equivalent 100 g^–1^ FW. The homogeneous juice was centrifuged at 10,000 × *g* for 10 min and the supernatant was filtered through a 0.45 μm Millipore filter and then injected into an HPLC system (Hewlett-Packard HPLC series 1100) to quantify individual sugars and organic acids, according to García-Pastor et al. (2020b). Individual sugars were detected by refractive index detector and organic acids by absorbance at 210 nm. Results were expressed as g 100 g^–1^ FW. For quantification, a standard curve of pure sugars and organic acids purchased from Sigma-Aldrich (Poole, United Kingdom) was used.

### Quality Parameters and Visual Decay Incidence During Storage

The firmness and colour of table grapes were measured at harvest and during postharvest storage. Firmness (N mm^–1^) was measured in 50 berries (10 berries per replicate) per treatment as the force that achieved a 5% deformation of the berry diameter, using a Texture Analyzer (TX-XT2i, Stable Microsystems, Godalming, Surrey, United Kingdom). Colour parameters [viz. L^∗^, a^∗^ b^∗^, Hue angle (arctan b/a)] were measured individually in another 50 berries from each treatment using a Minolta colorimeter (CRC200, Minolta Camera Co., Osaka, Japan).

Decay incidence was evaluated 5 days after being inoculated by *B. cinerea* and considered spoiled based on a visual scale of six hedonic points named as stages: S0, S1, S2, S3, S4, and S5, according to García-Pastor et al. (2020b), where: S0, without damage; S1, wound browning; S2, microbial growth covering 1–2 mm of the wound; S3, microbial growth covering 3–4 mm of the wound; S4, microbial growth covering 4–5 mm of the wound and even showing mycelial growth; S5, all the wound covered (6 mm) with the fungus and mycelium was observed. Results were expressed as the percentage of spoiled grapes in each stage based on the total number of fruits per box (mean ± SE of five replicates). Mean values ± SE per replicate were used for further statistical analysis.

### Statistical Analysis

A Student’s *t*-test (*p* < 0.05) was performed to detect significant differences between control and treatment (5 mM OA) samples at each given point during pre and postharvest stages. One-way ANOVA was used to determine the significance of mean differences among the three key developmental stages in preharvest (3DAT1, 3DAT2, and 3DAT3), postharvest storage time (0, 15, 30, and 45 days), and decay stages (S0–S5). HSD Duncan’s test was further used to examine if these differences were significant at *p* < 0.05. All statistical analyses were performed with SPSS software package v. 17.0 for Windows.

## Results

### Effect of Oxalic Acid Preharvest Treatment on Abscisic Acid, Relative *Vv*NCED1 Gene Expression, Abscisic Acid Catabolites, and Antioxidant Enzyme Activities During Berry Development

**FIGURE 1:**
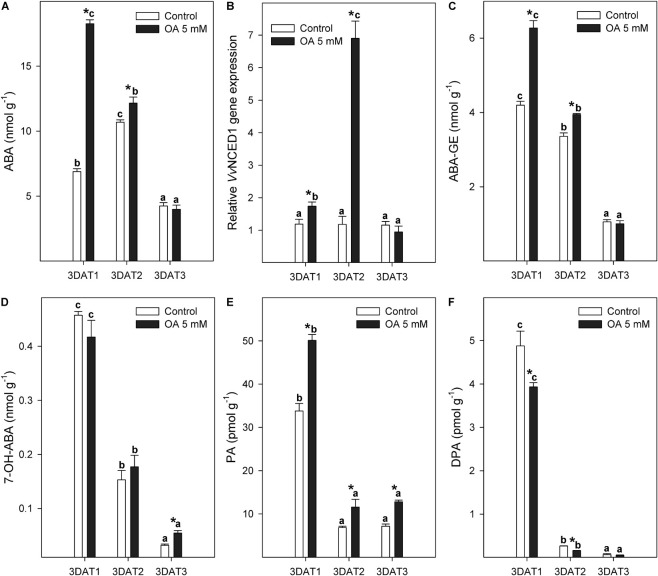
Evolution of ABA berry content (nmol g^–1^) **(A)**, relative *Vv*NCED1 gene expression **(B)**, and ABA catabolites [ABA-GE **(C)**, 7-OH-ABA **(D)**, PA **(E)**, and DPA **(F)**] content (nmol or pmol g^–1^) measured in control and 5 mM oxalic acid (OA)-treated ‘Magenta’ table grapes during three key growth and ripening stages in preharvest: 3DAT1 (3 days after the first treatment when berries reached ca. 40% of its final size), 3DAT2 (3 days after the second treatment when veraison stage started), and 3DAT3 (3 days after the third treatment, at harvest). Data are the mean ± SE. Significant differences (*p* < 0.05 according to Student’s *t*-test) between control and OA-treated berries were expressed as * symbol placed in the OA bar for each stage and parameter. Different lowercase letters show significant differences (*p* < 0.05 according to HSD Duncan’s test) among the three stages during berry development. *Vv*NCED1, *Vitis vinifera* 9-*cis*-epoxycarotenoid dioxygenase 1; ABA, abscisic acid; ABA–GE, ABA glucose ester; 7-OH-ABA, 7-hydroxy-ABA; PA, phaseic acid; DPA, dihydrophaseic acid.

Free ABA content peaked at the veraison stage (3DAT2) and decreased thereafter in control table grapes (Figure 1A). OA preharvest application caused a significant 2.7-fold increase in ABA content in treated berries at the 3DAT1 stage compared with control berries. Significant differences in ABA content between both treatments were also observed at the 3DAT2 stage, although to a lesser extent (Figure 1A). The relative expression level of *Vv*NCED1 was significantly upregulated by OA at 3DAT1 and 3DAT2 stages, but especially in the last one (veraison stage) with values sevenfold higher than in non-treated table grapes (Figure 1B), where molecular data did not proportionally support the biochemical data.

In relation to changes in ABA metabolism, the inactive glucose ester (ABA–GE) and three catabolites (7-OH-ABA, PA, and DPA) showed a significantly decreasing trend along the tree growth and ripening stages in both control and OA-treated berries, except for PA where no significant changes occurred in the last two stages (Figures 1C–F). Endogenous ABA–GE content was 49 and 18% higher in OA-treated table grapes than in control grapes at 3DAT1 and 3DAT2 stages, respectively (Figure 1C). 7-OH-ABA was the predominant ABA catabolite in grape berries (Figure 1D), followed by PA and DPA, which were considerably lower (Figures 1E,F). PA content in OA-treated table grapes was 1.5, 1.7, and 1.8-fold higher than in non-treated berries at 3DAT1, 3DAT2, and 3DAT3, respectively (Figure 1E).

**FIGURE 2:**
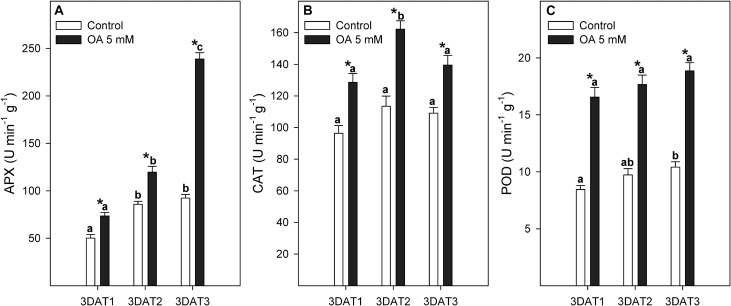
Evolution of APX **(A)**, CAT **(B)**, and POD **(C)** activities (U min^–1^ g^–1^) measured in control and 5 mM oxalic acid (OA)-treated ‘Magenta’ table grapes during three key growth and ripening stages in preharvest: 3DAT1 (3 days after the first treatment when berries reached ca. 40% of its final size), 3ADT2 (3 days after the second treatment when veraison stage started), and 3DAT3 (3 days after the third treatment, at harvest). Data are the mean ± SE. Significant differences (*p* < 0.05 according to Student’s *t*-test) between control and OA-treated berries were expressed as * symbol placed in the OA bar for each stage and parameter. Different lowercase letters show significant differences (*p* < 0.05 according to HSD Duncan’s test) among the three stages during berry development.

The antioxidant enzyme activity evolution during table grape development was dependent on the targeted enzyme (Figure 2). Thus, APX activity increased from 3DAT1 to 3DAT2 in control berries and then showed a stable behaviour. However, APX activity increased until the harvest date in OA-treated berries. The greatest effect of OA treatment on stimulating APX enzyme was observed at the 3DAT3 stage, its activity being 2.5-fold higher than non-treated berries (Figure 2A). Enzymatic activity of CAT was maintained during fruit development in non-treated berries, but an enzymatic peak of 1.4-fold higher activity was observed at the 3DAT2 or veraison stage in OA-treated table grapes (Figure 2B). Table grapes treated with OA showed a twofold increase in POD activity than in non-treated berries (Figure 2C).

### Effect of Oxalic Acid Preharvest Treatment on Bioactive Compound Content, Total Antioxidant Activity, and Berry Quality Parameters at Harvest

Berries treated with OA showed a significantly higher content of total anthocyanins and total phenols than the non-treated berries at harvest (Table 2). Specifically, those treated berries presented an approximately two-fold higher content of total anthocyanins in the whole fruit and skin tissue compared with control berries. In addition, both H-TAA and L-TAA significantly increased with OA treatment (Table 2).

**TABLE 2:** Bioactive compound content (total anthocyanins and total phenols), hydrophilic (H-TAA) and lipophilic (L-TAA) total antioxidant activity, individual anthocyanin content (dp-3-gluc; delphinidin 3-glucoside, cy-3-gluc; cyanidin 3-glucoside, pt-3-gluc; petunidin 3-glucoside, pn-3-gluc; peonidin 3-glucoside and mv-3-gluc; and malvidin 3-glucoside), total soluble solids (TSS), total acidity (TA), and individual sugar (glucose and fructose) and organic acid content (tartaric acid, malic acid, citric acid, ascorbic acid, succinic acid, and fumaric acid) in whole fruit (flesh + skin) or skin tissue of control and 5 mM OA-treated table grapes at harvest.

	Treatments	Flesh + skin	Skin
Total anthocyanins (mg 100 g^–1^)	Control	4.76 ± 0.38*^†^	16.69 ± 0.69*^†^
	OA 5 mM	9.48 ± 0.54*^†^	40.72 ± 1.96*^†^
Total phenols (mg 100 g^–1^)	Control	33.60 ± 1.23*^†^	158.18 ± 6.40*^†^
	OA 5 mM	43.84 ± 1.42*^†^	336.14 ± 25.35*^†^
TAA-hydrophilic (mg 100 g^–1^)	Control	179.11 ± 31.53*^†^	478.03 ± 25.11*^†^
	OA 5 mM	308.60 ± 13.00*^†^	962.96 ± 80.62*^†^
TAA-lipophilic (mg 100 g^–1^)	Control	31.80 ± 1.27*^†^	51.12 ± 1.30*^†^
	OA 5 mM	41.20 ± 1.42*^†^	71.37 ± 2.75*^†^
Dp-3-gluc (mg 100 g^–1^)	Control	0.80 ± 0.07^†^	1.35 ± 0.19*^†^
	OA 5 mM	0.93 ± 0.09^†^	3.46 ± 0.22*^†^
Cy-3-gluc (mg 100 g^–1^)	Control	0.16 ± 0.02*^†^	0.22 ± 0.07*^†^
	OA 5 mM	0.26 ± 0.06*^†^	0.57 ± 0.16*^†^
Pt-3-gluc (mg 100 g^–1^)	Control	0.32 ± 0.04*^†^	0.76 ± 0.19*^†^
	OA 5 mM	0.42 ± 0.06*^†^	1.55 ± 0.14*^†^
Pn-3-gluc (mg 100 g^–1^)	Control	1.78 ± 0.09*^†^	4.02 ± 0.56*^†^
	OA 5 mM	2.87 ± 0.07*^†^	7.14 ± 0.63*^†^
Mv-3-gluc (mg 100 g^–1^)	Control	2.05 ± 0.05*^†^	5.18 ± 0.54*^†^
	OA 5 mM	3.19 ± 0.07*^†^	8.80 ± 0.64*^†^
Total soluble solids (TSS) (g 100 g^–1^)	Control	16.90 ± 0.08*	–
	OA 5 mM	15.82 ± 0.06*	–
Total acidity (TA) (g 100 g^–1^)	Control	0.67 ± 0.01*	–
	OA 5 mM	0.73 ± 0.02*	–
Glucose (g 100 g^–1^)	Control	8.30 ± 0.13*	–
	OA 5 mM	7.80 ± 0.13*	–
Fructose (g 100 g^–1^)	Control	6.80 ± 0.11*	–
	OA 5 mM	6.39 ± 0.11*	–
Tartaric acid (g 100 g^–1^)	Control	0.34 ± 0.002*	–
	OA 5 mM	0.39 ± 0.010*	–
Malic acid (g 100 g^–1^)	Control	0.26 ± 0.007	–
	OA 5 mM	0.24 ± 0.013	–
Citric acid (g 100 g^–1^)	Control	0.07 ± 0.007	–
	OA 5 mM	0.06 ± 0.011	–
Ascorbic acid (g 100 g^–1^)	Control	0.0157 ± 0.0002*	–
	OA 5 mM	0.0166 ± 0.0002*	–
Succinic acid (g 100 g^–1^)	Control	0.00486 ± 0.00004*	–
	OA 5 mM	0.00520 ± 0.00001*	–
Fumaric acid (g 100 g^–1^)	Control	0.00054 ± 0.00002	–
	OA 5 mM	0.00051 ± 0.00008	–

Significant differences (*p* < 0.05 according to Student’s *t*-test) were expressed as * and ^†^ symbols placed in each column or row showing differences between both treatments and tissues, respectively, for each parameter at harvest.

Individual anthocyanin content was also significantly higher in OA-treated table grapes in both tissues, except delphinidin 3-glucoside content which only increased in the skin tissue (Table 2). All the functional parameters were significantly higher in the skin tissue rather than in the whole fruit (flesh + skin). The major anthocyanins of ‘Magenta’ table grapes were malvidin 3-glucoside and peonidin 3-glucoside in the same proportion, whose contents in the whole fruit and skin tissue were 1.6 and 1.7-fold higher in OA-treated table grapes than in control ones, respectively.

Total soluble solids and TA showed significant differences between both treatments at harvest (Table 2). OA-treated berries had 0.94-fold lower TSS and 1.10-fold higher TA than untreated grapes. Glucose and fructose content was significantly lower (by 6%) in OA-treated table grapes (Table 2), while those grapes had a significantly higher content of tartaric (by 13%), ascorbic (by 5%), and succinic acid (by 6.5%) than non-treated berries (Table 2).

### Effect of Oxalic Acid Preharvest Treatment on Quality Parameters, Berry Decay, Abscisic Acid and Catabolite Content, and Antioxidant Enzyme Activity During Postharvest Storage

**FIGURE 3:**
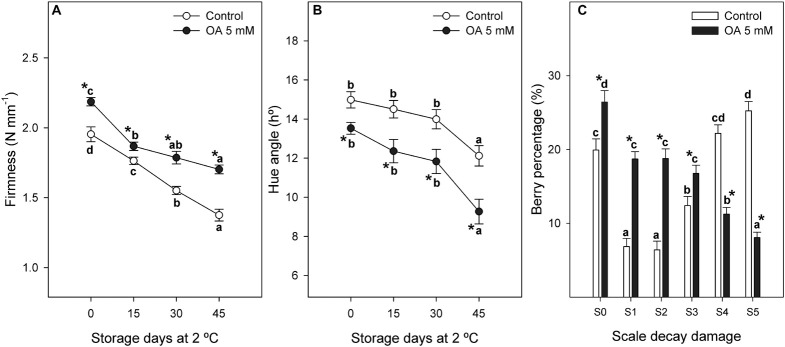
Evolution of firmness [N mm^–1^**(A)**] and hue angle colour [h° **(B)**] during 45 days of postharvest storage at 2°C of control and 5 mM OA-treated table grapes. Percentage of decayed berries [% **(C)**] according to the visual aspect scale (S0–S5) of *Botrytis cinerea* decay incidence in ‘Magenta’ table grapes as affected by preharvest treatments with control and oxalic acid (OA). Data are the mean ± SE. Significant differences (*p* < 0.05 according to Student’s *t*-test) between control and OA-treated berries were expressed as * symbol placed in the OA line or bar for each storage day or decay damage stage and parameter. Different lowercase letters show significant differences (*p* < 0.05 according to HSD Duncan’s test) among the storage days and decay damage stages in postharvest.

Table grapes treated with OA were 1.1-fold firmer than controls at harvest and differences between both treatments were maintained during cold storage (Figure 3A). Berry colour was also increased in OA-treated table grapes since significantly lower values of hue angle were observed in OA-treated berries than in control ones at harvest and during cold storage. Hue angle values were 1.3-fold lower for OA-treated berries than control ones at the end of postharvest storage (Figure 3B), showing that treated berries had a deeper purple colour than the untreated berries.

Oxalic acid preharvest treatment significantly reduced grey mould disease incidence in treated table grapes (Figure 3C). Thus, the percentage of berries without decay damage (S0) or with mild symptoms (S1, S2, and S3) was significantly higher in those berries treated with OA, and these treated berries showed a lower percentage of berries showing severe incidence (S4 and S5) than controls (Figure 3C).

**FIGURE 4:**
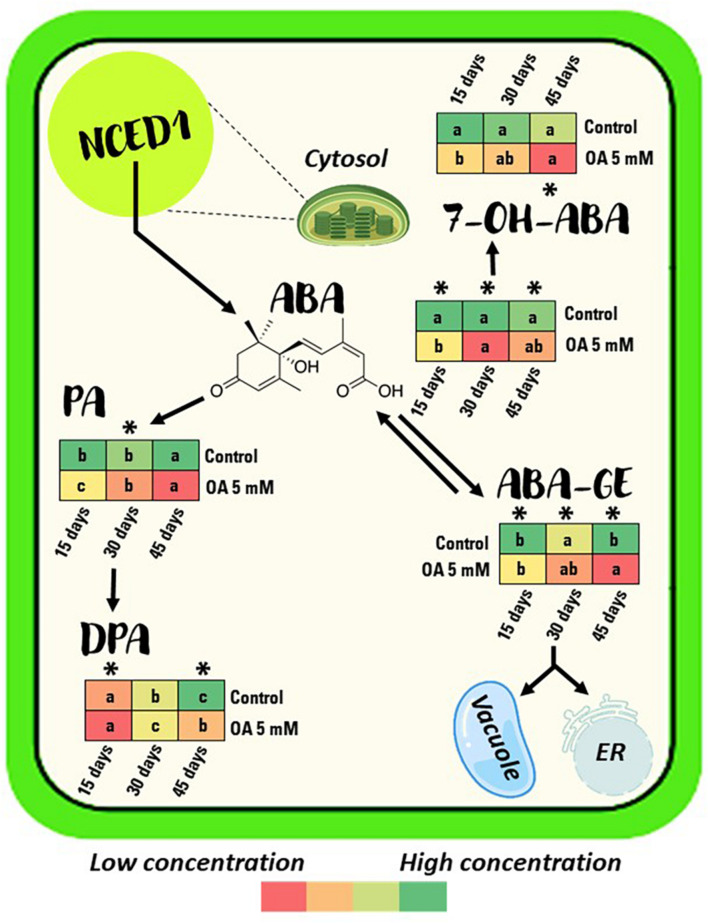
Berry content evolution of ABA and its catabolites content measured in control and 5 mM oxalic acid (OA)-treated ‘Magenta’ table grapes during 15, 30, and 45 days of postharvest storage at 2°C. Data are the mean ± SE. The colours in the diagram represent the low or high concentration, ranging from red to green, respectively. Significant differences (*p* < 0.05 according to Student’s *t*-test) were expressed as * symbol placed in each column showing differences between treatments for each storage day at 2°C. Different letters show significant differences (*p* < 0.05 according to HSD Duncan’s test) among storage days for each treatment. ABA, abscisic acid; ABA–GE, ABA glucose ester; 7-OH-ABA, 7-hydroxy-ABA; PA, phaseic acid; DPA, dihydrophaseic acid.

Oxalic acid-treated berries showed a significant, approximately 0.6- to 0.7-fold lower ABA content than control berries during 45 days of storage at 2°C (Figure 4 and Supplementary Table 1). Treated berries showed a significant 33, 23, and 58% lower ABA–GE content at the three sampling points, respectively, compared with non-treated berries (Figure 4 and Supplementary Table 1).

**FIGURE 5:**
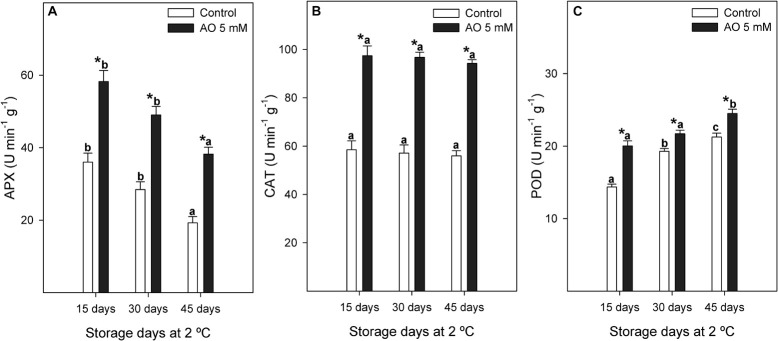
Evolution of ascorbate peroxidase [APX **(A)**], catalase [CAT **(B)**], and peroxidase [POD **(C)**] activities (U min^–1^ g^–1^) measured in control and 5 mM oxalic acid (OA)-treated ‘Magenta’ table grapes during 15, 30, and 45 days of postharvest storage at 2°C. Data are the mean ± SE. Significant differences (*p* < 0.05 according to Student’s *t*-test) between control and OA-treated berries were expressed as * symbol placed in the OA bar for each storage day and antioxidant enzyme. Different lowercase letters show significant differences (*p* < 0.05 according to HSD Duncan’s test) among the three storage days in postharvest.

Ascorbate peroxidase activity significantly increased by approximately 1.6 to 2-fold during postharvest storage by OA treatment (Figure 5A). The mean of CAT enzymatic activity from control berries was 57.21 ± 3.02 U min^–1^ g^–1^, whereas for OA-treated berries it was significantly higher, reaching an average of 96.14 ± 2.59 U min^–1^ g^–1^ (Figure 5B). POD enzyme showed an activity increase of 28, 11, and 13% at 15, 30, and 45 days, respectively, in OA-treated table grapes compared to controls (Figure 5C).

## Discussion

### Oxalic Acid Preharvest Treatment Upregulates Abscisic Acid Metabolism, Relative *Vv*NCED1 Gene Expression, and Antioxidant Enzyme Activity During Berry Development

Abscisic acid plays a key role in regulating several genes at the onset of veraison, including those involved in both anthocyanin signalling and biosynthesis pathway (Gambetta et al., 2010). Skin anthocyanin biosynthesis and accumulation start from veraison, as the onset of ripening, and continues until harvest (Costantini et al., 2015). The ABA accumulation was also accompanied by sugar accumulation and berry softening, which supports its role in controlling this hormone several ripening-associated processes in grapes (Wheeler et al., 2009; Fortes et al., 2015; Pilati et al., 2017). These processes are mainly under genetic control. Gene expression and activation of the biosynthetic enzymes are also influenced by climatic conditions and cultural practices, including the use of exogenous plant growth regulators (PGRs) (Yamamoto et al., 2015; Basile et al., 2018). The endogenous free ABA content is determined by the dynamic balance between biosynthesis and catabolism/conjugation. In our study, free ABA content of non-treated ‘Magenta’ seedless table grapes increased at the 3DAT2 or veraison stage following a decrease (Figure 1A), as reported by Torres et al. (2018). The OA treatment application significantly increased the ABA content at the 3DAT1 stage compared with the control grapes (Figure 1A), whereas its effect was maintained at the 3DTA2 or veraison stage, but to a lesser extent (Figure 1A). These biochemical changes may be supported by the upregulation of the *Vv*NCED1 gene by OA treatment at 3DAT1 and 3DAT2 stages (Figure 1B). However, the 2.7-fold increase of ABA content in OA-treated berries at 3DAT1 showed a lack of correlation with the twofold upregulation of *Vv*NCED1 expression and the ABA–GE or other catabolites content at this developmental stage. In contrast to the results obtained at 3DAT1, the relative *Vv*NCED1 gene expression showed a concomitant increment (≈ sevenfold increase) in OA-treated berries at the onset of ripening or 3DAT2 stage (Figure 1B). Thus, we suggest that the greatest ABA content in the OA-treated grapes at 3DAT1 (Figure 1A) began prior to the highest upregulation of endogenous ABA biosynthesis observed at 3DAT2, and was mediated by the relative expression of the rate-limiting *Vv*NCED1 gene (Figure 1B). In this sense, this increment on ABA levels could be supported by an ABA-translocation from leaves to berries, as a physiological plant response to OA preharvest treatment.

Table grapes treated with 5 mM of OA showed a significantly higher content of ABA–GE at 3DAT1 than non-treated berries (Figure 1C). ABA and ABA–GE were the dominant compounds in the ABA metabolic pool, and our results revealed that their concentration was significantly increased by 5 mM OA, particularly at 3DAT1 and 3DAT2 (Figures 1A,C). This suggests that the initial increases at 3DAT1 on ABA content (Figure 1A) by OA treatment may largely result from the increase mediated by ABA biosynthesis (Figure 1B). However, ABA concentrations reached near peak levels prior to the highest upregulation of the *Vv*NCED1 gene observed at 3DAT2 or the veraison stage, an ABA translocation also being possible from other parts of the vine, as was previously reported by others authors (Shiozaki et al., 1999; Keller et al., 2015). Specifically, Shiozaki et al. (1999) suggested that the transport of ABA from the leaves *via* phloem contributes to its increase in the grape berry during ripening. These authors reported that ABA accumulation in the grape berry during ripening was inhibited by vine defoliation at the veraison stage. On the contrary, free ABA in the phloem was not exported from berry to leaves. Moreover, demand-driven rise in phloem inflow at the beginning of the table grape ripening was necessary and sufficient to reverse a stress-induced berry shrinkage and enable sugar accumulation and berry growth under drought-stress conditions (Keller et al., 2015). OA treatment was applied by foliar spray on the vine, and maybe the *Vv*NCED1 gene expression of leaves could be also stimulated, leading to an increase in the ABA content at 3DAT1, which could be transported *via* the phloem to the berries as an exogenous import (Castellarin et al., 2016). Applications with ABA caused an increase in ABA content in both leaves and berries of ‘Malbec’ grapevine plants (Murcia et al., 2017), downregulating the relative expression level of *Vv*NCED1 in ABA-treated leaves, likely due to an ABA homeostasis. In this sense, Castellarin et al. (2016) have reported the increases in ABA, which occurred earlier in ‘Zinfandel’ grapes during their softening and prior to sharp increases in sugars and anthocyanins. In addition, these authors also observed an absence of significant expression of the *Vv*NCED during the accumulation in ABA content. However, these increases were coincident with the decline in the DPA catabolite, indicating that initial increases in ABA may result from lower catabolism and/or exogenous import. In relation to colour development, previous studies (Castellarin et al., 2016) showed that a disruption of phloem transport to berry clusters (induced through girdling), prior to the onset of ripening, completely inhibited colour development.

On the other hand, the seven-fold increase in relative *Vv*NCED1 gene expression of OA-treated berries at the 3DAT2 stage (Figure 1B) was not proportionally correlated with the 10% increase in ABA content at this developmental stage. Thus, the values of free ABA at the 3DAT2 developmental stage could be regulated by different metabolic pathways: (1) higher conjugation of ABA with glucose in treated berries, increasing ABA–GE content, which acts as a reservoir of ABA (Figure 1C); and (2) higher ABA catabolism at the position 8′, leading to a higher PA content, which is biologically active and serves as a bona fide ABA-like phytohormone. However, very similar levels of ABA–GE and ABA catabolites were observed at 3DAT2 between both treatments. Thus, although there is no available information reporting ABA translocation from table grape berries to other parts of the vine, including the leaves, it is highly probable, according to the reported metabolomic and molecular data, that OA preharvest treatment leads to a dynamic balance or redistribution of berry-derived ABA through the phloem into the aerial parts of the vine. Increasing evidence indicates that various plant membrane transport systems play a significant role in adaptation to stress conditions, such as drought. The role of various transport systems in ABA translocation, stomatal, cuticular, and root responses, and also osmotic adjustment has been summarised by Jarzyniak and Jasiński (2014). In contrast, other reports have shown that *Vv*NCED1 was upregulated by ABA and ethephon treatments (Pilati et al., 2017; Suehiro et al., 2019), explaining the intracellular ABA level measured by Pilati et al. (2017).

Our results describe the content of ABA catabolites (Figures 1C–F) and showed a decreasing trend in 7-OH-ABA, PA, and DPA during grape ripening, which was in agreement with those published by Torres et al. (2018). The catabolism mediated by the hydroxylation of 7′ and 8′-positions was induced in OA-treated table grapes at 3DAT3 or harvest. The OA treatment applied during berry development and ripening on-vine resulted in an accumulation of ABA content at the 3DAT1 stage, which was further supported at the molecular level by the upregulation of the *Vv*NCED1 gene expression. Similarly, OA also contributed to increasing levels of ABA–GE and PA catabolites during the growth and ripening cycle, which have been highlighted as key molecules in grapevine development and its physiological responses to environmental stresses (Castellarin et al., 2016).

During the stress response, plant cells exhibit defense mechanisms to detoxify the synthesised ROS including enzymes such as superoxide dismutase (SOD), CAT, POD, and APX. SOD converts O2^⋅–^ to H_2_O_2_, which could be finally eliminated by CAT, POD, and APX activities (Hodges et al., 2004). In general, these antioxidant enzymes showed higher activity at the three stages studied in table grapes from OA-treated vines than in controls. Increases in the activity of antioxidant enzymes have been also obtained after OA postharvest treatment in climacteric fruits (Zheng X. et al., 2007; Deng et al., 2015; Razzaq et al., 2015) and, recently, at preharvest treatment (Martínez-Esplá et al., 2019). The imbalance between the production and elimination of ROS, due to a decline in the activity of antioxidant enzymes, and an increase in lipoxygenase activity may partly be responsible for initiating the senescence in fruits (Hodges et al., 2004; Razavi and Hajilou, 2016). Our results suggest that preharvest application of 5 mM of OA could delay the senescence of treated table grapes during postharvest throughout the upregulation of these antioxidant systems during the developmental cycle and their maintenance during storage as addressed below.

### Oxalic Acid Preharvest Treatment Modulates Bioactive Compound Content, Total Antioxidant Activity, and Berry Quality Parameters at Harvest

In 2016 and 2017, OA was applied at 1, 5, and 10 mM concentrations and clusters were harvested when berries reached colour, size, and TSS content characteristic of this cultivar, according to commercial criteria. Given the fact that the ripening process is heterogeneous in the clusters within a vine, harvesting was performed on four different dates (Supplementary Figures 1A,B). For both growing cycles, 5 mM OA accelerated the on-vine berry ripening process since a higher amount of clusters were harvested from these OA-treated vines at the first harvest date, whereas no significant effects were observed for 1 or 10 mM concentrations. In addition, it is worth noting that accumulated yield at the last harvesting date was significantly higher in 5 mM OA-treated vines with respect to controls, whereas yield was not affected by 1 mM OA treatment and was reduced by 10 mM OA treatment. Thus, 5 mM OA was chosen as the best dose for the 2018 experiment, and similar results were obtained in terms of accelerating the on-vine ripening and increasing vine yield (Supplementary Figure 1C). The effects of 5 mM OA treatment on increasing vine yield were due to an enhanced berry volume, and the reduction in yield observed in 10 mM OA treatment was due to a lower berry size. In addition, in the ‘Magenta’ cultivar, the ripening process of the berries within a cluster is also heterogeneous, and then, berries that are not fully coloured are cut and discarded after harvesting by operators in the field. These poorly coloured berries were reduced in clusters from 5 mM treated vines, which also contributed to increasing vine yield, apart from the effect on increasing berry size.

On the other hand, our results showed that 5 mM OA preharvest treatment significantly increased the berry volume (mm^3^) by 11, 16, and 13.5% in the 2016, 2017, and 2018 seasons, respectively, than control berries (Supplementary Figure 2). The effect of OA preharvest treatment increasing berry size has also been reported in sweet cherry cultivars, ‘Sweet Heart’ and ‘Sweet Late’, by Martínez-Esplá et al. (2014), manifested by higher fruit volume and weight in cherries from treated trees than from controls, the higher effect being found with 2 mM OA. Berry size is widely acknowledged to affect berry quality. For instance, a negative relationship between TSS concentration and berry size has been described in *V. vinifera* L. cv. Syrah (clone SH1A) (Barbagallo et al., 2011). These authors observed that TSS decreased from the smallest to the largest berries that were divided into four categories based on. This effect was also found by Roby et al. (2004). However, no correlation was found between berry size and juice total acidity (TA). Our results showed a similar effect since 5 mM OA-treated berries had significantly higher volume than non-treated berries with the lowest TSS content (Supplementary Figure 2 and Table 2).

In general, our results show that the 5 mM OA-treated table grapes are berries with high bioactive molecule content and antioxidant potential at commercial harvest. Preharvest treatment of vines with OA at 5 mM led to significant increases in bioactive compound content such as total anthocyanins, total phenols, ascorbic acid, and TAA (H-TAA and L-TAA) at harvest in both whole fruit and skin tissue (Table 2). Treated table grapes also had higher individual anthocyanins content than the non-treated berries in both tissues (Table 2). In general, all these functional parameters were mainly improved in the skin tissue. Similar results inducing accumulation of phenolic and anthocyanins at harvest have also been found in other climacterics and non-climacteric fruits when OA was applied at pre- or postharvest stages (Valero et al., 2011; Martínez-Esplá et al., 2014, 2019; Deng et al., 2015; Razzaq et al., 2015; Razavi and Hajilou, 2016; Zhu et al., 2016; Kok and Bal, 2019; García-Pastor et al., 2020a), leading to fruits with higher antioxidant capacity. The mechanism by which OA increased the bioactive compounds and antioxidant properties is not well understood, although it could be attributed to the activation of phenylalanine ammonia-lyase (PAL) activity, the key enzyme in the phenyl propanoid pathway, which is involved in phenolic biosynthesis (Deng et al., 2015; Razavi and Hajilou, 2016; Martínez-Esplá et al., 2019). The higher content of total and individual anthocyanins in table grapes treated with OA could be related to endogenous ABA accumulation (Figure 1A), as reported by Keller et al. (2015) and Shahab et al. (2020). As expected, bioactive compound content, mainly individual anthocyanins, was higher in the skin than in the whole berry (Table 2). Anthocyanins are located in the berry skin, being responsible for skin colouration. Therefore, the content of these bioactive compounds is diluted when we analysed the whole berry (flesh + skin).

Finally, table grapes treated with OA 5 mM showed a significantly lower TSS content, mediated by lower glucose and fructose content, as well as higher tartaric, ascorbic, and succinic acid content, leading to higher TA as compared with untreated grapes (Table 2). Thus, it seems that OA 5 mM applied in preharvest delayed the ripening process of ‘Magenta’ table grapes, leading to berries with lower individual sugar concentration and higher acid content. Nevertheless, total and individual anthocyanin concentrations, responsible for berry colour and other important parameters related to ripening, were found at higher levels in grapes from OA-treated vines than in controls, which would indicate a more advanced ripening stage. The effect of OA treatment on berry ripening parameters was higher for colour and anthocyanin concentration than for TSS or sugar and acid content. As commented previously, clusters were harvested when berries reached their commercial ripening stage according to commercial criteria and they were in a similar ripening stage. However, analytical results show differences in some parameters related to ripening among control and treated berries, although in a different way depending on the considered parameter. Thus, ripening is a complex process and its evolution cannot be followed by a single parameter, the differences in TSS content possibly being related to the higher berry volume observed in OA-treated berries. On the other hand, OA preharvest treatment increased ABA accumulation at 3DAT1 and 3DAT2, which would lead to colour changes at veraison, as previously was reported when ‘Carménère’ clusters were sprayed with 50 and 100 μl L^–1^ ABA during preveraison (Villalobos-González et al., 2016), thereby contributing to accelerate berry on-vine ripening and increase anthocyanin content.

### Oxalic Acid Preharvest Treatment Influences Quality Parameters, Decay Berry Percentage, Abscisic Acid Metabolism, and Antioxidant Enzyme Activity During Postharvest Storage

Berry firmness at harvest was significantly higher in OA-treated table grapes than in controls (Figure 3A). Significant decreases occurred in firmness values during storage at 2°C, although the effect of OA delaying firmness losses were maintained during postharvest storage (Figure 3A). This OA long-lasting effect, which has also been reported elsewhere, delayed postharvest ripening by retarding softening in some climacteric fruits (Razavi and Hajilou, 2016; Zhu et al., 2016; Martínez-Esplá et al., 2019). Nevertheless, the effects of OA on delaying fruit softening process during storage could also be attributed to a reduction in the activity of cell wall hydrolytic enzymes, as has been reported for exo-polygalacturonase (exo-PG) and pectin methylesterase (PE) in OA postharvest-treated mango and plum fruits, respectively (Wu et al., 2011; Razzaq et al., 2015). In this sense, we hypothesize that this possible reduction on these two-cell wall hydrolytic enzymes, exo-PG and PE, could be in turn related to the higher activity of antioxidant enzymes mediated by OA treatment, which could delay the senescence process in the fruit (Figures 2A–C). Furthermore, the formation of oxalate–pectin as a result of OA treatment (Razzaq et al., 2015), leading to reinforcing wall structure of mesocarp cells and slowing down the softening process, could be associated with the effect of this treatment.

On the other hand, berry colour, expressed as hue angle, was improved at harvest and during postharvest cold storage in OA-treated table grapes (Figure 3B). This fact is related to the effect observed in Table 2 on the increase of total and individual anthocyanins content in both whole fruit and skin tissue. An increase in colour by OA treatments was also reported in climacteric and non-climacteric fruits (Martínez-Esplá et al., 2014; García-Pastor et al., 2020a). As previously commented, it has been suggested that OA promoted the accumulation of phenylpropanoid metabolites through the activation of the PAL enzyme. Contradictorily, no stimulation of this enzyme was observed by the OA treatment in a previous report (Zheng et al., 2012). According to our results, we hypothesise that OA could improve the berry colour throughout the ABA metabolomic stimulation, mediated at the same time by the homeostasis of its catabolites and transcribed by the *Vv*NCED1 gene.

Finally, a significantly lower incidence of *B. cinerea* was observed in berries from OA-treated vines than non-treated vines (Figure 3C). OA contributes to induce systemic resistance in plants, and that this may be due to both an increase in POD activity and synthesis of new POD isoforms, upregulating defence-related enzymes (Tian et al., 2006). Our results show that OA treatment resulted in significant changes in activities of defence enzymes such as POD (Figure 2C) and also other antioxidant enzymes (Figures 2A,B, 5A,B) during preharvest, at harvest, and during postharvest periods. On the other hand, disease resistance in fruit decreases during postharvest ripening as physiological and biochemical changes increase fruit susceptibility to pathogen infection, and the linkage between postharvest fruit ripening and increasing disease susceptibility is very strong. Thus, the effect of OA on decreasing fruit decay incidence might also be attributed to the delay of berry senescence, as has been reported by Zheng X. L. et al. (2007). Therefore, it should be highlighted that to elucidate this hypothesis, we measured the endogenous ABA and catabolite content during 15, 30, and 45 days of postharvest storage at 2°C (Figure 4 and Supplementary Table 1), and a significantly lower ABA and catabolite content in OA 5 mM-treated berries than in controls were found. OA-treated berries showed a less advanced stage of senescence than non-treated table grapes. Therefore, preharvest application of OA proved to be an effective method to delay quality deterioration and extend the storage shelf-life of table grapes, as was observed when it was applied as a postharvest treatment (Zheng et al., 2012).

Other PGRs, such as MeJa and salicylate derivatives (SA, ASA, and MeSa), applied as preharvest treatments, have been recently reported to affect the quality traits of ‘Magenta’ and ‘Crimson’ seedless table grape cultivars, although differently, depending on concentration (García-Pastor et al., 2019, 2020c). Thus, MeJa and salicylate derivatives applied at 5 and 10 mM delayed the berry ripening process and reduced crop yield in both cultivars, while ripening was accelerated and yield increased when these compounds were applied at 1, 0.1, and 0.01 mM concentrations. The effects of these treatments at high doses on reducing total yield were attributed to a delay or inhibition of the ripening process since many berries failed to ripen properly and some clusters did not reach the requested commercial quality. However, the concentration that showed better results in terms of berry maturity-quality, and bioactive compounds in the ‘Magenta’ seedless cultivar depended on the applied compound. Thus, 0.1 mM MeJa, 0.01 mM SA, 0.1 mM ASA, and 0.01 mM MeSa treatments, applied at key points of berry development, accelerated berry ripening, mainly colour evolution due to increased anthocyanin biosynthesis, as well as enhanced berry size, weight, firmness, TSS, and antioxidant bioactive compound content (phenolics and individual anthocyanins). Moreover, preharvest application of SA, ASA, and MeSA induced resistance of table grapes to be colonized with *B. cinerea*, which was attributed to the increased levels of phenolic compounds and the activity of antioxidant enzymes APX, CAT, and POD, (García-Pastor et al., 2020b). With respect to OA, in previous experiments performed during the growing seasons of 2016 and 2017, OA treatments were applied at 1, 5, and 10 mM, and the 5 mM concentration was chosen as the optimum among the three concentrations tested, in terms of yield, berry maturity-quality, and bioactive compounds (data not shown), to be applied in the present experiment (2018 season). In this study, OA preharvest treatment at 5 mM has shown similar effects in the ‘Magenta’ table grape as the other PGRs tested, improving its quality traits and inducing berry resistance to *B. cinerea* incidence. Specifically, in terms of colour, which is the main aim of these research studies, a significantly 1.3-fold increase in total anthocyanins content in the whole fruit (flesh + skin tissues) was observed by MeJa and SA treatments than untreated berries. However, greater results with 1.7 and 2-fold increases on this content were achieved by the application of ASA and MeSa or OA, respectively. Although in terms of improving total anthocyanins content in the skin tissue, where these coloured pigments are mainly synthesized and concentrated, MeSa preharvest treatment showed significantly lower effectiveness, increasing this content on OA (1.5-fold increase vs. 2.4-fold increase in MeSa and OA-treated berries skin, respectively, than control berries skin). Thus, OA preharvest treatment could be a more useful tool compared with other PGRs tested (MeJa, SA, ASA, or MeSa) in improving quality traits, mainly in terms of skin colour, of ‘Magenta’ poor-coloured table grapes.

## Conclusion

As an important signalling molecule, a preharvest application of OA at 5 mM improved the skin colour of table grapes at harvest by the upregulation of *Vv*NCED1 and ABA homeostasis during berry development and on-vine ripening. Additionally, OA delayed table grape postharvest ripening and senescence processes during storage, which was mainly mediated by the stimulation of enzymatic and non-enzymatic antioxidant systems, as well as by the reduction in the ABA metabolism. In conclusion, the preharvest application of 5 mM OA could be a useful tool to improve the colour and quality of poor-coloured table grape cultivars at harvest and during postharvest storage.

## Data Availability Statement

The original contributions presented in the study are included in the article/Supplementary Material, further inquiries can be directed to the corresponding author.

## Author Contributions

DV, MS, LT, MA, and PZ conceived and designed the work in association with other authors. FG and PZ performed the field treatments. MG-P performed most of the analytical determination in collaboration with MG and VS-E. MG-P and SG-M analysed the data. MG-P wrote the manuscript under the supervision of PZ and MA. All authors approved the final version of the manuscript.

## Conflict of Interest

The authors declare that the research was conducted in the absence of any commercial or financial relationships that could be construed as a potential conflict of interest.

## Publisher’s Note

All claims expressed in this article are solely those of the authors and do not necessarily represent those of their affiliated organizations, or those of the publisher, the editors and the reviewers. Any product that may be evaluated in this article, or claim that may be made by its manufacturer, is not guaranteed or endorsed by the publisher.
