# Supplementary material for: Oxalic Acid Preharvest Treatment Improves Colour and Quality of Seedless Table Grape ‘Magenta’ Upregulating on-Vine Abscisic Acid Metabolism, Relative VvNCED1 Gene Expression, and the Antioxidant System in Berries
Source: Front Plant Sci. 2021 Nov 1;12:740240. doi: 10.3389/fpls.2021.740240 (PMC8591251; doi:10.3389/fpls.2021.740240)
Supplement: Supplementary file 1 [file Data_Sheet_1.docx]

Supplementary Material

# Supplementary Figures and Tables

**Supplementary Figure 1.** Accumulated yield (kg vine^-1^) in control and OA treated vines in 2016 **(A)**, 2017 **(B)** and 2018 **(C)** experiments. Data are the mean ± SE of five replicates of three vines (15 vines) for each treatment in ‘Magenta’ cultivar. Different lowercase letters show significant differences (*p* < 0.05 according to HSD Duncan’s test) among the treatments for each harvest date in 2016 and 2017 seasons. Significant differences (*p* < 0.05 according to Student’s *t*-test) between control and OA-treated berries were expressed as * symbol placed in the OA bar for each harvest date in 2018 season.

**Supplementary Figure 2.** Effect of vine OA treatments on berry volume (mm^3^) in 2016 **(A)**, 2017 **(B)** and 2018 **(C)** experiments. Data are the mean ± SE of five replicates of 30 berries (10 berries from each vine) from the first harvest date for both treatments in ‘Magenta’ cultivar. Different lowercase letters show significant differences (*p* < 0.05 according to HSD Duncan’s test) among the treatments for 2016 and 2017 seasons. Significant differences (*p* < 0.05 according to Student’s *t*-test) between control and OA-treated berries were expressed as * symbol placed in the OA bar for 2018 season.

**Supplementary Table 1.** Berry content evolution’s supplementary data of ABA and its catabolites content (nmol or pmol g^-1^) measured in control and 5 mM oxalic acid (OA)-treated ‘Magenta’ table grapes during 15, 30 and 45 days of postharvest storage at 2 ºC. Data are the mean ± SE. Significant differences between treatments for each storage day at 2 ºC (*p* < 0.05 according to Student’s t-test) were highlighted in **Figure 4**. ABA: abscisic acid; ABA-GE: ABA glucose ester; 7-OH-ABA: 7- hydroxy-ABA; PA: phaseic acid; DPA: dihydrophaseic acid.

|  | |  | | **ABA** | **ABA-GE** | **7-OH-ABA** | **PA** | **DPA** |
| --- | --- | --- | --- | --- | --- | --- | --- | --- |
|  | |  | | **(nmol g^-1^)** | **(nmol g^-1^)** | **(nmol g^-1^)** | **(pmol g^-1^)** | **(pmol g^-1^)** |
| **Control** | **15 days** | | 1.96 ± 0.11 | | 1.13 ± 0.04 | 0.03 ± 0.002 | 0.01 ± 0.001 | 0.06 ± 0.004 |
|  | **30 days** | | 1.73 ± 0.09 | | 0.86 ± 0.06 | 0.03 ± 0.002 | 0.009 ± 0.001 | 0.10 ± 0.007 |
|  | **45 days** | | 1.81 ± 0.15 | | 1.38 ± 0.10 | 0.02 ± 0.001 | 0.005 ± 0.001 | 0.20 ± 0.015 |
| **OA 5 mM** | **15 days** | | 1.40 ± 0.07 | | 0.76 ± 0.05 | 0.02 ± 0.002 | 0.008 ± 0.001 | 0.04 ± 0.003 |
|  | **30 days** | | 0.99 ± 0.09 | | 0.66 ± 0.02 | 0.02 ± 0.002 | 0.006 ± 0.0004 | 0.09 ± 0.005 |
|  | **45 days** | | 1.20 ± 0.09 | | 0.58 ± 0.04 | 0.01 ± 0.002 | 0.004 ± 0.0004 | 0.06 ± 0.005 |
